# Tai Chi for Essential Hypertension

**DOI:** 10.1155/2013/215254

**Published:** 2013-08-06

**Authors:** Jie Wang, Bo Feng, Xiaochen Yang, Wei Liu, Fei Teng, Shengjie Li, Xingjiang Xiong

**Affiliations:** ^1^Department of Cardiology, Guang'anmen Hospital, China Academy of Chinese Medical Sciences, Beixiange 5, Xicheng District, Beijing 100053, China; ^2^School of Life Sciences, Tsinghua University, Beijing 100084, China

## Abstract

*Objectives*. To assess the current clinical evidence of Tai Chi for essential hypertension (EH). *Search Strategy*. 7 electronic databases were searched until 20 April, 2013. *Inclusion Criteria*. We included randomized trials testing Tai Chi versus routine care or antihypertensive drugs. Trials testing Tai Chi combined with antihypertensive drugs versus antihypertensive drugs were also included. *Data Extraction and Analyses*. Study selection, data extraction, quality assessment, and data analyses were conducted according to the Cochrane standards. *Results*. 18 trials were included. Methodological quality of the trials was low. 14 trials compared Tai Chi with routine care. 1 trial compared Tai Chi with antihypertensive drugs. Meta-analysis all showed significant effect of TaiChi in lowering blood pressure (BP). 3 trials compared Tai Chi plus antihypertensive drugs with antihypertensive drugs. Positive results in BP were found in the other 2 combination groups. Most of the trials did not report adverse events, and the safety of Tai Chi is still uncertain. *Conclusions*. There is some encouraging evidence of Tai Chi for EH. However, due to poor methodological quality of included studies, the evidence remains weak. Rigorously designed trials are needed to confirm the evidence.

## 1. Introduction

Hypertension is a significant medical and public health issue which puts an enormous burden on health care resources and the community [1]. It is a chronic medical condition in which the systemic arterial blood pressure (BP) is elevated. Serious complications including cardiovascular and cerebrovascular diseases would be preventable if the rise in BP with age could be prevented or diminished [2]. The majority of hypertensive patients require long-term treatment. However, effective treatment of essential hypertension (EH) is limited by availability, cost, and adverse effects of conventional western medicine treatment [3]. Thus, a certain proportion of the population, especially in Asia, has turned to complementary and alternative medicine (CAM), including traditional Chinese medicine (TCM), in searching for a treatment modality with potential efficacy and few adverse effects [4–9]. For seeking the best evidence of TCM in making decisions for hypertensive patients, an increasing number of systematic reviews (SRs) and meta-analysis have been conducted to assess the efficiency and safety of TCM for EH [10–14]. It is demonstrated that, as an effective adjunct treatment, TCM could contribute to lowing BP and relieving hypertension-related symptoms for EH. 

Tai Chi (also known as Tai Chi Quan or Shadow Boxing), which originated in ancient China, is a Chinese conditioning exercise well known for its graceful movement. It has been practiced for centuries in the East for health promotion and longevity. In recent years, there has been a growing interest and prevalence in Tai Chi exercise in Western societies [15, 16]. During the practice, it combines deep diaphragmatic breathing with continuous body motions to achieve a harmonious balance between body and mind. Previous researches have indicated that Tai Chi exercise may improve health-related fitness (including cardiorespiratory function, muscular strength, balance, and flexibility), quality of life, and psychological well-being. Recent studies also suggest that it may have beneficial effects for patients with cardiovascular conditions and some cardiovascular risk factors, including hypertension [17–19]. It is found out that Tai Chi could contribute to low BP smoothly and improve symptoms and signs especially [19–21]. And the efficacy of Tai Chi for treating hypertension is suggested by a large number of published case series and randomized trials [20–24]. Currently, Tai Chi used alone or combined with antihypertensive drugs has been widely used as an alternative and effective method for the treatment of EH worldwide. However, it has not been evaluated according to the PRISMA systematic review standard. This study aims to assess the current clinical evidence of Tai Chi for EH.

## 2. Methods

### 2.1. Database and Search Strategies

Literature searches were conducted in the following 7 electronic databases: Chinese National Knowledge Infrastructure (CNKI) (1980–2013), Chinese Scientific Journal Database (VIP) (1989–2013), Chinese Biomedical Literature Database (CBM) (1978–2013), Wanfang data (1998–2013), Cochrane Library (January, 2013), EMBASE (1980–2013), and PubMed (1959–2013). We also searched the reference list of retrieved papers. As Tai Chi is mainly practiced and studied in China, four major databases in Chinese were searched to retrieve the maximum possible number of trials of Tai Chi for EH. All of those searches were ended on 20 April, 2013. Ongoing registered clinical trials were searched in the website of Chinese clinical trial registry (http://www.chictr.org/en/) and international clinical trial registry by U.S. national institutes of health (http://clinicaltrials.gov/). The following search terms were used individually or combined: “hypertension,” “essential hypertension,” “primary hypertension,” “blood pressure,” “Tai Chi,” “T'ai Chi,” “Tai Chi Quan,” “Taijiquan,” “Shadow Boxing,” “clinical trial,” and “randomized controlled trial”. The bibliographies of included studies were searched for additional references. 

### 2.2. Inclusion Criteria

Only patients with EH could be involved in this review, which is diagnosed by systolic blood pressure (SBP) ≥140 mmHg, or, diastolic blood pressure (DBP) ≥90 mmHg. We included all the parallel randomized controlled trials (RCTs) testing Tai Chi used alone versus antihypertensive drugs, routine care, or other exercise in patients with hypertension. RCTs testing Tai Chi combined with antihypertensive drugs versus antihypertensive drugs were included as well. There were no restrictions on population characteristics, language, and publication type. The main outcome measure was BP. Duplicated publications reporting the same groups of participants were excluded. 

### 2.3. Data Extraction and Quality Assessment

Two authors conducted the literature searching (X. J. Xiong, S. J. Li), study selection (X. J. Xiong, W. Liu), and data extraction (X. J. Xiong, B. Feng) independently. The extracted data included authors, title of study, year of publication, study size, age and sex of the participants, study characteristics, diagnosis standard, details of methodological information, treatment process, details of the intervention and control, outcomes, and adverse effects for each study. Disagreement was resolved by discussion and reached consensus through a third party (J. Wang). 

The criteria from the Cochrane Handbook for Systematic Review of Interventions, Version 5.1.0 (X. J. Xiong, F. Teng) were used to assess the methodological quality of trials independently [43]. The items included the followings 7 aspects: random sequence generation (selection bias), allocation concealment (selection bias), blinding of participants and personnel (performance bias), blinding of outcome assessment (detection bias), incomplete outcome data (attrition bias), selective reporting (reporting bias), and other biases. The quality of all the included trials was categorized to low/unclear/high risk of bias (“Yes” for a low of bias, “No” for a high risk of bias, “Unclear” otherwise). Then trials were categorized into three levels: low risk of bias (all the items were in low risk of bias), high risk of bias (at least one item was in high risk of bias), and unclear risk of bias (at least one item was in unclear). 

### 2.4. Data Synthesis

We used the Revman 5.1 software provided by the Cochrane Collaboration for data analyses. Dichotomous data were presented as risk ratio (RR) and continuous outcomes as mean difference (MD) or weight mean difference (WMD), both with 95% confidence interval (CI). Heterogeneity was recognized significant when *I* ^2^ ≥ 50%. Fixed effects model was used if there is no significant heterogeneity of the data; random effects model was used if significant heterogeneity existed (50% < *I* ^2^ < 85%). Publication bias would be explored by funnel plot analysis if sufficient studies were found.

## 3. Result

### 3.1. Description of Included Trials

**Figure 1:**
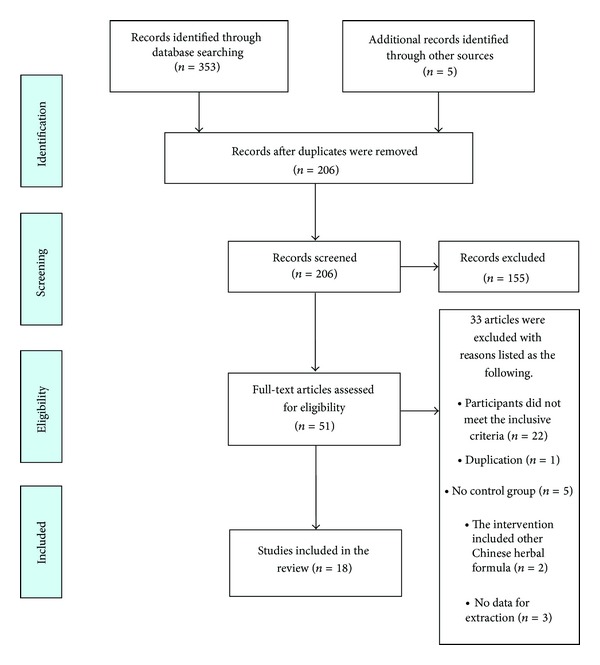
PRISMA 2009 flow diagram.

As shown in Figure 1, the flow chart depicted the search process and study selection. After primary searches from the above 7 electronic databases, 353 articles were retrieved: CNKI (*n* = 161), VIP (*n* = 71), CBM (*n* = 46), Wanfang data (*n* = 21), Cochrane Library (*n* = 5), Pubmed (*n* = 17), and EMBASE (*n* = 32). 206 articles were screened after 152 duplicates were removed. After reading the subjects and abstracts, 155 articles were excluded. Full texts of 51 articles were retrieved, and 33 articles were excluded with reasons listed as below: participants did not meet the inclusive criteria (*n* = 22), duplication (*n* = 1), no control group (*n* = 5), the intervention included other Chinese herbal formulae (*n* = 2), and no data for extraction (*n* = 3). Finally, 18 RCTs [25–42] were included. 16 RCTs of them were published in Chinese [25–33, 35–41]; 1 RCT was published in English [34]; 1 RCT was published in Korean [42]. The characteristics of included trials were listed in Table 1. 

1371 patients with EH were included. There was a wide variation in the age of subjects (35–75 years). 18 trials specified six diagnostic criteria of hypertension, five trials [27, 29, 32, 33, 39] used Chinese Guidelines for the Management of Hypertension-2005 (CGMH-2005), four trials [26, 28, 36, 37] used 1999 WHO-ISH guidelines for the management of hypertension (1999 WHO-ISH GMH), one trial [30] used Chinese Guidelines for the Management of Hypertension-1999 (CGMH-1999), one trial [38] used the Seventh Report of the Joint National Committee on Prevention, Detection, Evaluation, and Treatment of High Blood Pressure (JNC-7), one trial [42] used the Sixth Report of the Joint National Committee on Prevention, Detection, Evaluation, and Treatment of High Blood Pressure (JNC-6), and six trials [25, 31, 34, 35, 40, 41] only demonstrated patients with EH without specific information about diagnostic standard. 

Interventions included all the exercises based on Tai Chi alone (including 12-type Tai Chi, 24-type Tai Chi, 48-type Tai Chi, Yang-type Tai Chi, and Chen-type Tai Chi) or combined with antihypertensive drugs. The controls included routine care (including walking, slow-running, and aerobics) or antihypertensive drugs alone. Four trials [29, 32, 41, 42] investigated Tai Chi using alone versus routine care. Six trials [25–27, 30, 35, 38] investigated 24-type Tai Chi using alone versus routine care. One trial [37] investigated 48-type Tai Chi using alone versus routine care. One trial [34] investigated Yang-type Tai Chi using alone versus routine care. One trial [33] investigated 24-type Tai Chi, 48-type Tai Chi, and Yang-type Tai Chi together versus routine care. One trial [40] investigated 12-type Tai Chi using alone versus routine care. One trial [31] investigated Tai Chi using alone versus antihypertensive drugs (reserpine or compound rutin tablets). One trial [28] investigated Tai Chi combined with cilazapril versus cilazapril. One trial [36] investigated 24-type Tai Chi combined with cilazapril versus cilazapril. One trial [39] investigated Chen-type Tai Chi combined with nifedipine versus nifedipine.

The total treatment duration ranged from 2 to 60 months. The variable exercises of Tai Chi are presented in Table 1. All of the 18 trials used the BP as the outcome measure. Adverse effect was also described. 

### 3.2. Methodological Quality of Included Trials

The majority of the included trials were assessed to be of general poor methodological quality according to the predefined quality assessment criteria (as shown in Table 2). The randomized allocation of participants was mentioned in all trials; however, only 2 trials stated the methods for sequence generation with stratified sampling [26, 30]. The remaining 16 trials [25, 27–29, 31–42] did not mention the concrete random sequence generation at all. Insufficient information was provided to judge whether or not it was conducted properly. Allocation concealment, blinding of participants and personnel, and blinding of outcome assessment were not mentioned in all trials. 2 trials [26, 34] reported drop-out. The rest of trials [25, 27–42] have not reported it at all. None of trials had a pretrial estimation of sample size. One trial [26] mentioned 5-year follow-up. We tried to contact the author by telephone, fax, email, and other ways for further detailed information about the trials; however, no information could be got until now. 

### 3.3. Effect of the Interventions

All the included trials [25–42] compared Tai Chi used alone or combined with antihypertensive drugs with routine care or antihypertensive drugs. A change in BP was reported in all the RCTs. According to the different intervention strategies, it could be divided into the following subgroups. 

#### 3.3.1. Tai Chi versus Routine Care

As mentioned above, there were 5 types of Tai Chi used in this review, including 12-type Tai Chi, 24-type Tai Chi, 48-type Tai Chi, Yang-type Tai Chi, and Chen-type Tai Chi. Therefore, we combined all these types together for comprehensive analysis. 14 trials comparing Tai Chi with routine care were included [25–27, 29, 30, 32–35, 37, 38, 40–42]. Among them, 4 trials [25, 32, 35, 37] used three classes to evaluate treatment effects on BP: significant effective (DBP decreased by 10 mmHg reaching the normal range, or, DBP has not yet returned to normal but has been reduced ≥20 mmHg), effective (DBP decreased to less than 10 mmHg reaching the normal range, or, DBP decreased by 10–19 mmHg, but did not reach the normal range, or, SBP decreased ≥30 mmHg), and ineffective (Not to meet the above standards). The trial showed significant difference in favor of the Tai Chi group as compared to routine care group (RR: 3.39 [1.81, 6.34]; *P* = 0.0001) (Table 3).

When it comes to SBP, 10 trials [17, 27, 29, 30, 33, 34, 38, 40–42] showed heterogeneity in the results. Thus, random-effects model was used for statistical analysis. The meta-analysis showed there is significant beneficial effect on the Tai Chi group as compared to routine care group (WMD: −12.43 [−12.62, −12.24]; *P* < 0.00001) (Table 4). 

When it comes to DBP, 10 trials [17, 27, 29, 30, 33, 34, 38, 40–42] showed heterogeneity in the results. Thus, random-effects model was used for statistical analysis. The meta-analysis showed that there is significant beneficial effect on the Tai Chi group as compared to routine care group (WMD: −6.03 [−6.16, −5.90]; *P* < 0.00001) (Table 5).

#### 3.3.2. Tai Chi versus Antihypertensive Drugs (Western Medicine)

1 trial [31] investigated Tai Chi using alone versus antihypertensive drugs (reserpine or compound rutin tablets). When it comes to SBP, it showed no applicable heterogeneity in the result. Thus, fixed-effects model was used for statistical analysis. The meta-analysis showed that there is significant beneficial effect on the Tai Chi group as compared to antihypertensive drugs group (WMD: −14.30 [−14.31, −14.29]; *P* < 0.00001) (Table 4). 

When it comes to DBP, it also showed no applicable heterogeneity in the result. Thus, fixed-effects model was used for statistical analysis. The meta-analysis showed that there is significant beneficial effect on the Tai Chi group as compared to antihypertensive drugs group (WMD: −6.00 [−6.01, −5.99]; *P* < 0.00001) (Table 5).

#### 3.3.3. Tai Chi plus Antihypertensive Drugs versus Antihypertensive Drugs (Western Medicine)

3 trials [28, 36, 39] investigated Tai Chi combined with antihypertensive drugs versus antihypertensive drugs. Among them, 1 trial [36] used three classes to evaluate treatment effects on BP. The trials showed no significant difference between Tai Chi plus antihypertensive drugs group and antihypertensive drugs (cilazapril) group (RR: 2.50 [0.69, 9.06]; *P* = 0.16) (Table 3). 

When it comes to SBP, 2 independent trials [28, 39] showed significant heterogeneity in the results. Thus, random-effects model was used for statistical analysis. The meta-analysis showed that there are significant beneficial effects on the Tai Chi plus antihypertensive drugs group as compared to antihypertensive drugs group (WMD: −9.34 [−10.89, −7.79]; *P* < 0.00001) (Table 4). 

When it comes to DBP, 2 independent trials [28, 39] also showed significant heterogeneity in the results. Thus, random-effects model was used for statistical analysis. The meta-analysis showed that there are significant beneficial effects on the Tai Chi plus antihypertensive drugs group as compared to antihypertensive drugs group (WMD: −7.16 [−7.71, −6.60]; *P* < 0.00001) (Table 5).

### 3.4. Publication Bias

The number of trials was too small to conduct any sufficient additional analysis of publication bias. 

### 3.5. Adverse Effect

Only 1 trial mentioned the adverse effect [30]. The other 17 trials [25–29, 31–42] did not report it at all. No specific symptoms and signs were found about Tai Chi in the trial. 

## 4. Discussion

Currently, with increasing concern about long-term medication and the potential adverse effects of antihypertensive drugs [44–46], nondrug therapy and natural herbal products have gained more and more popularity by hypertensive patients worldwide [47–54]. As a special form of exercise, Tai Chi has made great contributions to the healthcare and well-being of the people for its unique advantages in preventing and curing diseases, especially in China. And until now, more and more researches have been conducted to explore the health-enhancing qualities of Tai Chi for various cardiovascular diseases (CVDs) and cerebrovascular diseases [17–24]. It has become an effective mean of secondary prevention of CVDs. It is found that Tai Chi could not only contribute to lowing BP smoothly, recovering the heart function, reversing cardiovascular risk factors, but also improving symptoms and quality of life (QOL) [55–57]. Although there are 2 SRs about Tai Chi on lowering resting blood pressure (including hypertension, acute myocardial infarction, older people with chronic conditions, healthy elderly men, middle-aged women, and other diseases) [52, 57], the role of Tai Chi for EH is still unknown due to different inclusion criteria and search strategies. Therefore, this paper aims to assess the current clinical evidence of Tai Chi for EH. 

This systematic review included 18 randomized trials with 1371 hypertensive patients. As compared to routine care groups, positive results in SBP (WMD: −12.43 [−12.62, −12.24]; *P* < 0.00001), DBP (WMD: −6.03 [−6.16, −5.90]; *P* < 0.00001), and BP (RR: 3.39 [1.81, 6.34]; *P* = 0.0001) were found in Tai Chi group, indicating that BP could be improved, and SBP and DBP could be decreased by 12.43 mmHg and 6.03 mmHg, respectively, after Tai Chi treatment. As compared to antihypertensive drugs (reserpine or compound rutin tablets) group, positive results in SBP (WMD: −14.30 [−14.31, −14.29]; *P* < 0.00001) and DBP (WMD: −6.00 [−6.01, −5.99]; *P* < 0.00001) were found in Tai Chi group, indicating that SBP and DBP could be decreased by 14.30 mmHg and 6.00 mmHg, respectively, after Tai Chi treatment. As compared to antihypertensive drugs groups, there is no difference between Tai Chi plus cilazapril group and cilazapril group in BP (RR: 2.50 [0.69, 9.06]; *P* = 0.16), indicating that no more beneficial effect was found in the combination therapy; however, positive results in SBP (WMD: −9.34 [−10.89, −7.79]; *P* < 0.00001) and DBP (WMD: −7.16 [−7.71, −6.60]; *P* < 0.00001) were found in the other 2 Tai Chi plus antihypertensive drugs groups, indicating that SBP and DBP could be decreased by 9.34 mmHg and 7.16 mmHg, respectively, after the combination therapy. In conclusion, except cilazapril treatment group, BP was improved in the other subgroups, and a significant decrease in both SBP and DBP was found. Recently, it is confirmed by many studies that a small reduction in BP may result in a large reduction in the risk of stroke and myocardial infarction [58]. What is more, a reduction of 5 mmHg in SBP has been associated with a 7% reduction in all-cause mortality [59]. Based on the paper and meta-analyses of the outcome on either SBP or DBP, Tai Chi may have positive effects for BP. Our review showed that SBP and DBP could be decreased by 9.34–14.30 mmHg and 6.00–7.16 mmHg, respectively, indicating that Tai Chi could not only reduce BP, but also have potential protective effect on reducing the risk of cardiovascular and cerebrovascular diseases. It is worth noting that the cardiovascular protective effect of Tai Chi is closely related to the long-term adherence to regular exercise. In this review, the total treatment duration ranged from 2 to 60 months. In particular, Han et al., 2010, [26] conducted a 5-year follow-up trial, showing that Tai Chi is helpful to control the hypertension and release tension emotion in order to improve QOF in middle-aged and elderly patients with EH. 

However, although positive results were found in this meta-analysis, the encouraging clinical evidence of Tai Chi for EH might be weakened due to the small sample size and poor methodological qualities of included trials. And the positive findings should be interpreted conservatively. Firstly, the methodological quality of the included RCTs is assessed to be generally low. All trials included in this paper had risk of bias in terms of design, reporting, and methodology. They provided only inadequate reporting of study design, allocation sequence, and allocation concealment. Thus, potential selection bias might be generated. Randomization was mentioned but without further details in most trials, which do not allow a proper judgment of the conduction of the RCTs. It could not rule out the possibility that declared RCTs may not be really randomized. Both blinding of participants and personnel and blinding of outcome assessment have not been used due to the difficulty of operation. Thus, potential performance bias and detection bias might be generated. Drop-out was only reported in 2 trials [26, 34]. The majority of trials have not reported it at all. None of trials had a pretrial estimation of sample size. Most of the included trials were not multicenter, large scale RCTs. If poorly designed, results would show larger differences as compared to the well designed trials, and the credibility about the conclusions will be greatly reduced. 

Secondly, adverse effects are not highly valued in most of the included trials. As we know that, safety is the basis for medication. However, it is always ignored and should be given priority in TCM [60–63]. In our review, only 1 trial reported the adverse effect of Tai Chi, and no adverse effect was found [30]. Most of the trials [25–29, 31–42] did not report it at all. Therefore, a definite conclusion about the safety of Tai Chi cannot be made clearly. It needs to be monitored rigorously and reported appropriately in the future clinical trials. 

Thirdly, the primary goal of treatment for EH is to reduce the mortality or prevent progression to severe complications [64]. Only one trial [26] reported the 5-year follow-up of Tai Chi. It was found out that there are 2 patients died of cerebral hemorrhage in the control group. However, there were no serious cardiovascular and cerebrovascular events in Tai Chi group. The outcomes from most of the included trials are mainly BP. Thus, there is a lack of definite data from all the trials on clinically relevant outcomes such as the mortality and incidence of complications. Clinical pieces of evidence of the efficacy of TCM on the mortality and morbidity of hypertension need to be strengthened in future researches.

In summary, there is some encouraging evidence of Tai Chi for lowering BP in hypertensive patients, but the evidence remains weak due to poor methodological quality of included studies. Rigorously designed trials seem to be warranted to confirm the results.

## Figures and Tables

**Table 1:** Characteristics and methodological quality of included studies.

Study ID	Sample	Diagnosis standard	Intervention	Control	Course	Outcome measure
Wei et al. 2003 [25]	46	Unclear	24-type Tai Chi	routine care	12 m	BP
Han et al. 2010 [26]	60	1999 WHO-ISH GMH	24-type Tai Chi	routine care	60 m	BP
Wang et al. 2011 [27]	30	CGMH-2005	24-type Tai Chi	routine care	12 w	BP
Tang 2009 [28]	32	1999 WHO-ISH GMH	Tai Chi + control	cilazapril (25 mg qd)	6 m	BP
Chen et al. 2011 [29]	441	CGMH-2005	Tai Chi	routine care	24 m	BP
Mao and Sha 2006 [30]	62	CGMH-1999	24-type Tai Chi	routine care	8 w	BP
Yi et al. 1990 [31]	20	Unclear	Tai Chi	reserpine (4 mg tid), or compound rutin tablets (20 mg tid)	18 m	BP
Chen 2011 [32]	61	CGMH-2005	Tai Chi	routine care	12 m	BP
He et al. 2011 [33]	49	CGMH-2005	24-type Tai Chi, 48-type Tai Chi, and Yang-type Tai Chi	routine care	20 w	BP
Lo et al. 2012 [34]	58	Unclear	Yang-type Tai Chi	routine care	8 w	BP
Wang 2007 [35]	46	Unclear	24-type Tai Chi	routine care	8 m	BP
Luo 2006 [36]	84	1999 WHO-ISH GMH	24-type Tai Chi + control	cilazapril (2.5–5.0 mg qd)	6 m	BP
Wang et al. 2007 [37]	84	1999 WHO-ISH GMH	48-type Tai Chi	routine care	6 m	BP
Zhou 2007 [38]	120	JNC-7	24-type Tai Chi	routine care	12 w	BP
Chen and Lv 2006 [39]	40	CGMH-2005	Chen-type Tai Chi + control	nifedipine (50–100 mg qd)	10 w	BP
Song and Yu 2011 [40]	50	Unclear	12-type Tai Chi	routine care	2 m	BP
Wang et al. 2011 [41]	60	Unclear	Tai Chi	routine care	16 w	BP
Lee 2004 [42]	28	JNC-6	Tai Chi	routine care	6 w	BP

m: month; w: week.

**Table 2:** Quality assessment of included randomized controlled trials.

Included trials	Random sequence generation	Allocation concealment	Blinding of participants and personnel	Blinding of outcome assessment	Incomplete outcome data	Selective reporting	Other sources of bias	Risk of bias
Wei et al. 2003 [25]	Unclear	Unclear	Unclear	Unclear	Yes	No	Unclear	High
Han et al. 2010 [26]	Stratified sampling	Unclear	Unclear	Unclear	Yes	No	Unclear	Unclear
Wang et al. 2011 [27]	Unclear	Unclear	Unclear	Unclear	Yes	No	Unclear	High
Tang 2009 [28]	Unclear	Unclear	Unclear	Unclear	Yes	No	Unclear	High
Chen et al. 2011 [29]	Unclear	Unclear	Unclear	Unclear	Yes	No	Unclear	High
Mao and Sha 2006 [30]	Stratified sampling	Unclear	Unclear	Unclear	No	No	Unclear	Unclear
Yi et al. 1990 [31]	Unclear	Unclear	Unclear	Unclear	Yes	No	Unclear	High
Chen 2011 [32]	Unclear	Unclear	Unclear	Unclear	Yes	No	Unclear	High
He et al. 2011 [33]	Unclear	Unclear	Unclear	Unclear	Yes	No	Unclear	High
Lo et al. 2012 [34]	Unclear	Unclear	Unclear	Unclear	Yes	No	Unclear	High
Wang 2007 [35]	Unclear	Unclear	Unclear	Unclear	Yes	No	Unclear	High
Luo 2006 [36]	Unclear	Unclear	Unclear	Unclear	Yes	No	Unclear	High
Wang et al. 2007 [37]	Unclear	Unclear	Unclear	Unclear	Yes	No	Unclear	High
Zhou 2007 [38]	Unclear	Unclear	Unclear	Unclear	Yes	No	Unclear	High
Chen and Lv 2006 [39]	Unclear	Unclear	Unclear	Unclear	Yes	No	Unclear	High
Song and Yu 2011 [40]	Unclear	Unclear	Unclear	Unclear	Yes	No	Unclear	High
Wang et al. 2011 [41]	Unclear	Unclear	Unclear	Unclear	Yes	No	Unclear	High
Lee 2004 [42]	Unclear	Unclear	Unclear	Unclear	Yes	No	Unclear	High

**Table 3:** Analyses of blood pressure.

Trials		Intervention (*n*/*N*)	Control (*n*/*N*)	RR [95% CI]	*P* value
*Tai Chi versus routine care *					
24-type Tai Chi versus routine care	1	20/23	14/23	4.29 [0.98, 18.72]	0.05
Tai Chi versus routine care	1	25/31	15/30	4.17 [1.33, 13.07]	0.01
24-type Tai Chi versus routine care	1	20/23	13/23	5.13 [1.18, 22.24]	0.03
48-type Tai Chi versus routine care	1	36/42	32/42	1.18 [0.61, 5.74]	0.27
*Meta-Analysis *	4	101/119	74/118	3.39 [1.81, 6.34]	0.0001
*Tai Chi plus antihypertensive drugs versus antihypertensive drugs *					
24-type Tai Chi plus cilazapril versus cilazapril	1	40/44	32/40	2.50 [0.69, 9.06]	0.16
*Meta-Analysis *	1	40/44	32/40	2.50 [0.69, 9.06]	0.16

**Table 4:** Analyses of systolic blood pressure.

Trials		WMD [95% CI]	*P* value
*Tai Chi versus routine care *			
24-type Tai Chi versus routine care	1	−10.50 [−10.86, − 10.14]	<0.00001
24-type Tai Chi versus routine care	1	−12.36 [−14.76, − 9.96]	<0.00001
Tai Chi versus routine care	1	−8.48 [−8.82, − 8.14]	<0.00001
24-type Tai Chi versus routine care	1	−24.42 [−26.18, − 22.66]	<0.00001
24/48/Yang-type Tai Chi versus routine care	1	−18.30 [−19.37, − 17.23]	<0.00001
Yang-type Tai Chi versus routine care	1	−4.34 [−5.20, − 3.48]	<0.00001
24-type Tai Chi versus routine care	1	−18.20 [−18.54, − 17.86]	<0.00001
12-type Tai Chi versus routine care	1	−15.92 [−18.56, − 13.28]	<0.00001
Tai Chi versus routine care	1	−12.97 [−15.10, − 10.84]	<0.00001
Tai Chi versus routine care	1	−17.60 [−23.44, − 11.76]	<0.00001
*Meta-analysis *	10	−12.43 [−12.62, − 12.24]	<0.00001
*Tai Chi versus antihypertensive drugs *			
Tai Chi versus antihypertensive drugs (reserpine or compound rutin tablets)	1	−14.30 [−14.31, − 14.29]	<0.00001
*Meta-analysis *	1	−14.30 [−14.31, − 14.29]	<0.00001
*Tai Chi plus antihypertensive drugs versus antihypertensive drugs *			
Tai Chi plus cilazapril versus cilazapril	1	−7.60 [−9.24, − 5.96]	<0.00001
Chen-type Tai Chi plus nifedipine versus nifedipine	1	−24.00 [−28.75, − 19.25]	<0.00001
*Meta-analysis *	2	−9.34 [−10.89, − 7.79]	<0.00001

**Table 5:** Analyses of diastolic blood pressure.

Trials		WMD [95% CI]	*P* value
*Tai Chi versus routine care *			
24-type Tai Chi versus routine care	1	−3.70 [−4.89, − 2.51]	<0.00001
24-type Tai Chi versus routine care	1	−5.07 [−5.26, − 4.88]	<0.00001
Tai Chi versus routine care	1	−4.06 [−4.34, − 3.78]	<0.00001
24-type Tai Chi versus routine care	1	−11.18 [−11.67, − 10.69]	<0.00001
24/48/Yang-type Tai Chi versus routine care	1	−9.10 [−9.44, − 8.76]	<0.00001
Yang-type Tai Chi versus routine care	1	−1.20 [−3.57, 1.17]	0.32
24-type Tai Chi versus routine care	1	−6.90 [−7.92, − 5.88]	<0.00001
12-type Tai Chi versus routine care	1	−5.04 [−6.69, − 3.39]	<0.00001
Tai Chi versus routine care	1	−7.20 [−9.39, − 5.01]	<0.00001
Tai Chi versus routine care	1	−11.70 [−12.56, − 10.84]	<0.00001
*Meta-analysis *	10	−6.03 [−6.16, − 5.90]	<0.00001
*Tai Chi versus antihypertensive drugs *			
Tai Chi versus antihypertensive drugs (reserpine or compound rutin tablets)	1	−6.00 [−6.01, − 5.99]	<0.00001
*Meta-analysis *	1	−6.00 [−6.01, − 5.99]	<0.00001
*Tai Chi plus antihypertensive drugs versus antihypertensive drugs *			
Tai Chi plus cilazapril versus cilazapril	1	−7.07 [−7.63, − 6.51]	<0.00001
Chen-type Tai Chi plus nifedipine versus nifedipine	1	−10.60 [−14.11, − 7.09]	<0.00001
*Meta-analysis *	2	−7.16 [−7.71, − 6.60]	<0.00001
